# Chronic disseminated candidiasis in a patient with acute leukemia - an illustrative case and brief review for clinicians

**DOI:** 10.1186/s12879-024-09172-9

**Published:** 2024-03-06

**Authors:** Allison Graeter, Dasom Lee, Guy Handley, Aliyah Baluch, Olga Klinkova

**Affiliations:** 1 Department of Internal Medicine, University of South Florida, 17 Davis Blvd., Suite 308, 33606 Tampa, FL USA; 2 Division of Hematology, Stanford University, 94305 Stanford, CA USA; 3 Department of Infectious Disease and International Medicine, 1 Tampa General Circle, 33606 Tampa, FL USA; 4 Infectious Disease Division, Moffitt Cancer Center, 12902 USF Magnolia Drive, 33612 Tampa, FL USA

**Keywords:** Chronic disseminated candidiasis, Hepatosplenic candidiasis, Immunocompromised, Hematopoietic stem cell transplant, Neutropenia, Neutropenic fever

## Abstract

Chronic disseminated candidiasis (CDC) is a severe but rarely seen fungal infection presenting in patients with hematologic malignancies after a prolonged duration of neutropenia. A high index of suspicion is required to diagnose CDC as standard culture workup is often negative. While tissue biopsy is the gold standard of diagnosis, it is frequently avoided in patients with profound cytopenias and increased bleeding risks. A presumptive diagnosis can be made in patients with recent neutropenia, persistent fevers unresponsive to antibiotics, imaging findings of hypoechoic, non-rim enhancing target-like lesions in the spleen and liver, and mycologic evidence. Here, we describe the case of an 18-year-old woman with relapsed B-cell acute lymphoblastic leukemia treated with re-induction chemotherapy who subsequently developed CDC with multi-organ involvement. The diagnosis was made based on clinical and radiologic features with positive tissue culture from a skin nodule and hepatic lesion. The patient was treated for a total course of 11 months with anti-fungal therapy, most notably amphotericin B and micafungin, and splenectomy. After initial diagnosis, the patient was monitored with monthly CT abdomen imaging that showed disease control after 5 months of anti-fungal therapy and splenectomy. The diagnosis, treatment, and common challenges of CDC are outlined here to assist with better understanding, diagnosis, and treatment of this rare condition.

## Background

*Candida* species are colonizers of the human skin and gastrointestinal (GI) tract, existing as part of the microbiome in up to 60% of healthy individuals [1, 2]. Patients with hematologic malignancies are at increased risk for invasive candidiasis due to iatrogenic immunosuppression as well as chemotherapy- induced injury of the gastrointestinal mucosa that can lead to a translocation of the commensal *Candida species* and other gastrointestinal pathogens into a bloodstream [1].

There are two forms of invasive candidiasis that include candidemia and deep-seated tissue candidiasis [3, 4]. The latter arises from either hematogenous spread of the infection or from direct inoculation of the organism into a sterile tissue site [1, 3]. Chronic disseminated candidiasis (CDC) is one form of invasive candidiasis that occurs in patients with hematologic malignancies after a period of prolonged neutropenia upon neutrophil count recovery [1, 3]. The diagnosis of CDC is made based on the combination of clinical, radiographic, and laboratory data.

As microbiologic data is often negative, high index of suspicion is needed to make this diagnosis in the appropriate host. Here, we present a case of an 18-year-old patient with B- cell acute lymphoblastic leukemia (B-ALL) undergoing reinduction chemotherapy who developed CDC and describe classic radiographic and histopathologic manifestations of this condition as well as common challenges in diagnosis and treatment.

## Case presentation

An 18-year-old female patient with relapsed B-ALL was admitted for re-induction chemotherapy under Children’s Oncology Group protocol AALL1131, a regimen including vincristine, mitoxantrone, dexamethasone and intrathecal methotrexate; pegaspargase was omitted due to prior history of intracranial hemorrhage. Her induction course was complicated by port cellulitis treated with port removal, cefepime, and intravenous vancomycin. For neutropenic prophylaxis, she received intravenous micafungin 100 mg daily and oral acyclovir 400 mg twice daily. During admission, she developed neutropenic fever with repeatedly negative cultures. The chemistry panel revealed mildly elevated AST(aspartate aminotransferase) and ALT (alanine aminotransferase) at 55 units/L and 148 units/L respectively (ALT normal range < 35 units/L, AST normal range 14–36 units/L) for which an abdominal ultrasound was obtained demonstrating hepatomegaly with mild hepatic steatosis. Due to persistent neutropenic fever, prophylactic micafungin was transitioned to voriconazole 6 mg/kg twice daily for 2 loading doses, followed by 4 mg/kg twice daily.

On day 38 of treatment, the patient developed a left lower extremity skin nodule. Laboratory values demonstrated absolute neutrophil count of 400 cells/µL, AST of 67 units/L, ALT of 51 units/L, and alkaline phosphatase of 211 units/L (normal range 38–126 units/L). Repeat abdominal ultrasound demonstrated interval development of multiple hypoechoic splenic foci suspicious for microabscesses. Voriconazole was switched to intravenous liposomal amphotericin B (AmBisome ®) at 5 mg/kg daily. Skin punch biopsy of the left lower extremity was performed and tissue fungal culture revealed *Candida tropicalis* growth susceptible to fluconazole, caspofungin, and voriconazole; minimal inhibitory concentration values were not available. Diagnosis of CDC was made based on the constellation of the patient’s symptoms, splenic lesions on imaging, and presence of the nodular skin lesion with *Candida tropicalis*. At that point, antifungal therapy was changed to fluconazole per susceptibility testing. The patient’s fevers resolved, and she was discharged home.

She was admitted again to receive blinatumomab as part of her anti-neoplastic regimen. The course was again complicated by non-neutropenic fevers with negative microbiologic workup. However, an interim abdominal ultrasound performed 4 weeks after initial imaging demonstrated new hepatic target lesions and increased number of splenic lesions. Fluconazole was changed to intravenous micafungin 100 mg daily for suspected progression of CDC.

**Fig. 1:**
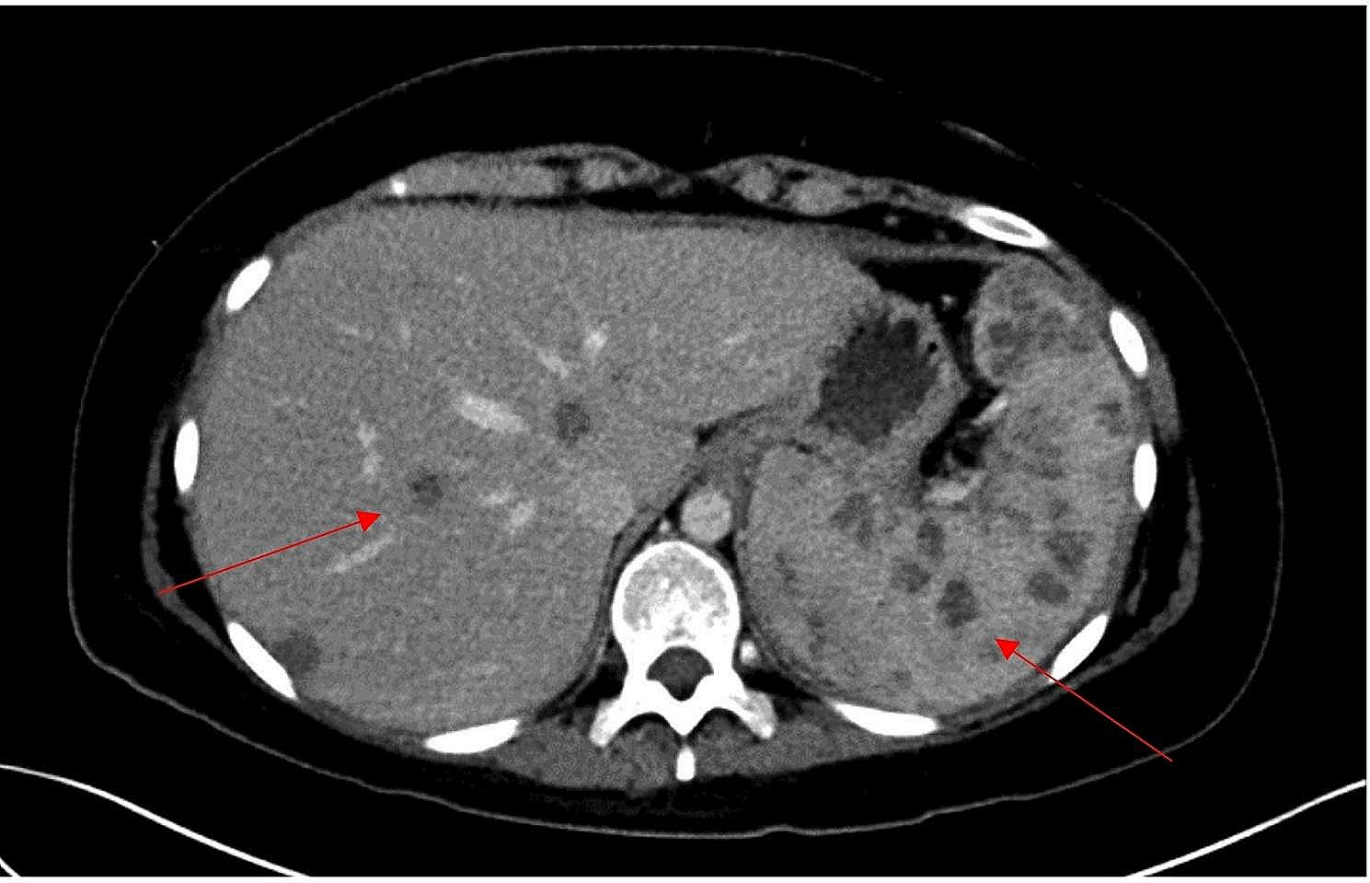
Multiple hypoenhancing lesions (red arrows pointing) in the liver and spleen shown in CT scan of thorax and abdomen with contrast

**Fig. 2:**
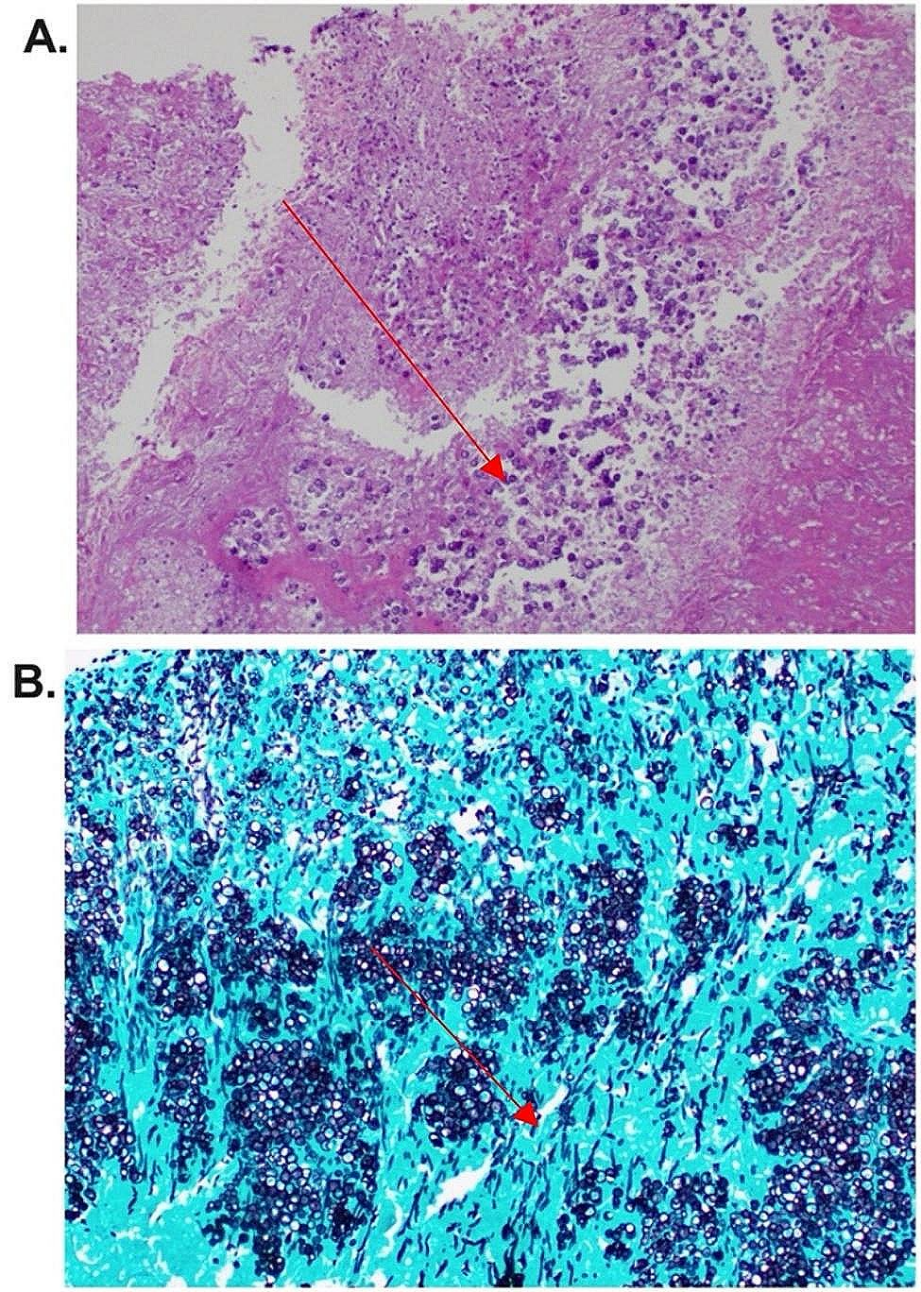
Liver lesion pathologic images. **(A)** Periodic Acid-Schiff stain showing extensive necrotizing granuloma (red arrow pointing) due to fungal infection and **(B)** Grocott’s Methenamine Silver stain showing mixture of hyphae, pseudohyphae, and yeast (red arrow pointing)

Shortly after, the patient was referred to our institution for allogeneic stem cell transplantation evaluation (SCT) for the treatment of her underlying hematologic condition. Pre-transplant imaging revealed multiple hepatosplenic lesions (Fig. 1), right kidney lesions, and a nodular appearing opacity in the lateral left lower lobe. Lung nodule biopsy revealed mild interstitial fibrosis and chronic inflammation without granulomas, with cultures negative for bacterial and fungal growth. The hepatic lesion was biopsied and revealed budding yeast consistent with *Candida* species (Fig. 2), although culture failed to grow this organism. Liver tissue specimen was sent for next generation 16 S rRNA sequencing (University of Washington, USA) and returned positive for *C. tropicalis*, confirming the diagnosis of persistent CDC. The patient was treated with dual antifungal therapy with intravenous micafungin 150 mg daily, (dose increased from prior) and intravenous liposomal amphotericin B 5 mg/kg daily. Splenectomy was performed 6 months after initial CDC diagnosis to reduce the burden of the disease. Follow up CT scans of the abdomen 1 month after splenectomy demonstrated stable to mildly improved hepatic lesions and stable right kidney lesions. She was continued on amphotericin B and micafungin throughout pre- and post-transplant periods for the total of 6 months following splenectomy. The patient ultimately underwent allogeneic SCT and passed away 32 days later after developing early post-SCT multi-organ failure unrelated to CDC that had been diagnosed 11 months earlier.

## Discussion

Here we describe a case of CDC with multi-organ involvement, highlighting the common challenges of diagnosis and treatment in severely immunocompromised patients. In a study of 292 patients with acute myelogenous leukemia, incidence of suspected CDC was 3.8%, with 72.7% of those patients developing the infection during or after induction therapy, and a 36.4% 90-day mortality [5].

The pathophysiology of CDC in immunocompromised patients is poorly understood but thought to be related to chemotherapy-induced mucositis and profound neutropenia. This allows for *Candida* species to enter the bloodstream where the organisms can translocate into the portal venous system and seed to the liver and spleen, which explains why the lesions are primarily seen in these two organs [1, 6]. Renal, pulmonary, and other organ involvement have also been documented but not as common. Immune reconstitution syndrome in settings of neutrophil recovery plays an important role in the pathogenesis of CDC such as contributing to the formation of granulomas as a result of inflammatory response as well as development of symptoms arising most commonly during or after neutrophil recovery [6].

The most common presentation of CDC is persistent or recurrent fevers despite neutrophil recovery and broad-spectrum antibiotic treatment. Right upper quadrant abdominal pain, fatigue, nausea, and hepatosplenomegaly are other common symptoms. The initial diagnosis of CDC is oftentimes presumptive and based on the clinical presentation, elevated liver enzymes and radiographic imaging with demonstration of multiple liver and/or spleen lesions.

On CT scan, MRI, or ultrasound imaging, abscesses appear as multiple, peripherally located, small and target-like lesions [7–9]. Blood culture work-up is often negative for *Candida* species growth, but history of candidemia within two weeks prior to CDC diagnosis can provide an additional clue in the appropriate clinical scenario. In the study by Pagano et al., only 32% of the patients with CDC had concurrent candidemia [10], and in the study by Chen et al., only 21% did [7]. Due to the low yield of blood culture work-up, other markers such as (1, 3)-β-D glucan can assist with the diagnosis of probable CDC [11], however, the exact specificity and sensitivity of these assays specifically for the diagnosis of CDC is unclear.

In certain cases, such as when the alternative diagnosis is in question, organism resistance is considered, or poor clinical response to therapy is observed, ultrasound or computer guided- needle aspiration or a biopsy of a lesion can be performed. Special stains, such as the Gomori Methenamine Silver (GMS) and Periodic Acid Schiff (PAS) stains, are used to visualize fungal forms seen as hyphal or pseudohyphal elements [12]. Based on limited reported data, the yield of tissue biopsy culture is low and was reported to be only 42% by Thaler et al. with even lower sensitivity in patients receiving antifungal therapy [13].

The criteria for the proven and probable invasive fungal diseases (IFDs), including invasive candidiasis, were recently updated in 2020 and published by the European Organization for Research and Treatment of Cancer and the Mycoses Study Group Education and Research Consortium (EORTC/MSGERC) [14]. Proven invasive fungal disease is diagnosed based on evidence of yeast forms on microscopic analysis, culture from sterile sample material, or amplification of fungal DNA by PCR combined with DNA sequencing from formalin-fixed paraffin-embedded tissue [14]. Probable invasive fungal disease, including invasive candidiasis, can be suspected and diagnosed based on host factors, clinical features, radiographic findings, and mycological evidence such as elevated (1, 3)- β- D glucan or T2Candida assay [14].

Once CDC is diagnosed, prompt initiation of anti-fungal therapy is recommended with either an echinocandin or a lipid formulation of amphotericin B at 3–5 mg/kg daily for at least two weeks. This is followed by step-down therapy with fluconazole 6 mg/kg daily until imaging reveals resolution of lesions, and finally maintenance therapy in patients with ongoing immunosuppression [15]. The treatment of fluconazole-resistant isolates should be guided by susceptibility testing. In patients with persistent fevers and abdominal symptoms despite appropriate antifungal therapy, a course of systemic glucocorticoids is recommended and has been shown to effectively control symptoms [15, 16]. In patients with extensive splenic involvement or large splenic abscesses, splenectomy may be considered to reduce the burden of disease, although this approach remains controversial [17]. Treatment of hematologic disease, including chemotherapy and hematopoietic cell transplantation, should not be delayed in patients with stable and controlled CDC [15].

In the case presented here, the diagnosis of CDC was made based on clinical and radiologic features with positive tissue culture from a skin nodule and hepatic lesion, even in the setting of antifungal use. The persistence of the patient’s lesions is likely explained by the nature of CDC rather than antifungal resistance. CDC can cause formation of granulomas that entrap yeast elements and lead to poor penetration of antifungal agents. In addition, fluctuating neutrophil counts, dysfunctional bone marrow production, and immunosuppression secondary to chemotherapy likely contributed to disease persistence. After achieving remission of B-ALL with neutrophil count recovery, splenectomy, and continued antifungal treatment with amphotericin B, the patient’s candidiasis was controlled as evidenced by decreasing size of hepatic lesions on CT imaging. Once CDC was controlled with long-term amphotericin B as demonstrated by repeat scans, the patient was able to successfully proceed with allogeneic HSCT.

In summary, here we present a case of disseminated *C. tropicalis* infection and highlight the difficulty of diagnosis of this potentially deadly disease due to oftentimes negative culture work-up. It is imperative that providers remain aware of possible CDC in immunosuppressed patients with persistent fevers, especially after neutrophil count recovery and immune reconstitution. If CDC is suspected based on the clinical picture, prompt treatment must be initiated per guidelines without delay.

## Data Availability

Not applicable.

## Abbreviations

CDC: Chronic disseminated candidiasis
GI: gastrointestinal
B-ALL: precursor B cell acute lymphoblastic leukemia
ANC: absolute neutrophil count
HSCT: hematopoietic stem cell transplant
CXR: chest x-ray
IDSA: Infectious Diseases Society of America
*C. tropicalis*: *Candida tropicalis*
